# The exercise pressor reflex and active O_2_ transport in peripheral arterial disease

**DOI:** 10.14814/phy2.14243

**Published:** 2019-10-22

**Authors:** Jon Stavres, Christopher T. Sica, Cheryl Blaha, Michael Herr, Jianli Wang, Samuel Pai, Aimee Cauffman, Jeffrey Vesek, Qing X. Yang, Lawrence I. Sinoway

**Affiliations:** ^1^ Penn State Heart and Vascular Institute Pennsylvania State University College of Medicine Milton S. Hershey Medical Center Hershey Pennsylvania; ^2^ Department of Radiology Pennsylvania State University College of Medicine Milton S. Hershey Medical Center Hershey Pennsylvania; ^3^ Milton S. Hershey Medical Center, Department of Molecular Biology Pennsylvania State University College of Medicine Hershey Pennsylvania; ^4^ Department of Neurosurgery Pennsylvania State University College of Medicine Milton S. Hershey Medical Center Hershey Pennsylvania

**Keywords:** ischemia, muscle oxygenation, occlusive disease, oxygen uptake

## Abstract

It is unclear if the exaggerated exercise pressor reflex observed in peripheral arterial disease (PAD) patients facilitates Oxygen (O_2_) transport during presymptomatic exercise. Accordingly, this study compared O_2_ transport between PAD patients and healthy controls during graded presymptomatic work. Seven PAD patients and seven healthy controls performed dynamic plantar flexion in the bore of a 3T MRI scanner. Perfusion, T_2_* (an index of relative tissue oxygenation), and SvO_2_ (a measure of venous oxygen saturation) were collected from the medial gastrocnemius (MG) during the final 10 seconds of each stage. Blood pressure was also collected during the final minute of each stage. As expected, the pressor response to presymptomatic work (4 kg) was exaggerated in PAD patients compared to controls (+14 mmHg ± 4 and +7 mmHg ± 2, *P* ≤ 0.034). When normalized to changes in free water content (S_0_), T_2_* was lower at 2 kg in PAD patients compared to controls (−0.91 Δms/ΔAU ± 0.3 and 0.57 Δms/ΔAU ± 0.3, *P* ≤ 0.008); followed by a greater increase in perfusion at 4 kg in the PAD group (+18.8 mL/min/100g ± 6.2 vs. −0.21 mL/min/100g ± 3.2 in PAD and controls, *P ≤ *0.026). Lastly, SvO_2_ decreased at 4 kg in both groups (−13% ± 4 and −2% ± 4 in PAD and controls, *P* ≤ 0.049), suggesting an increase in O_2_ extraction in the PAD group. Based on these findings, O_2_ transport appears to be augmented during graded presymptomatic work in PAD patients, and this may be partially mediated by an exaggerated pressor response.

## Introduction

Peripheral arterial disease (PAD) is a systemic vascular disease affecting approximately 8.5 million people in the United States and 200 million people worldwide (Roger et al., 2011; Shu and Santulli, 2018). The classic manifestation of PAD is pain in the lower legs during light to moderate intensity work (termed intermittent claudication, or IC), caused by an insufficient increase in oxygen (O_2_) delivery relative to demand. While regular exercise has been shown to improve PAD symptoms (Gardner et al., 2000), impaired O_2_ delivery limits exercise tolerance. This leads to a “vicious cycle,” where decreased levels of activity accelerate disease progression. Therefore, it is important to understand how the mechanisms of O_2_ transport and utilization are altered in PAD patients, as this can have implications for treatment and rehabilitation.

It is well established that convective O_2_ transport is impaired in PAD patients, and more recent evidence suggests that impaired O_2_ utilization at the mitochondria (and therefore diffusive O_2_ transport) may also contribute to the pathophysiology of PAD (Pipinos et al., 2003; AlGhatrif et al., 2017; Schmidt et al., 2017). This introduces some uncertainty regarding the sequence of disease progression; specifically, whether or not mitochondrial dysfunction occurs independent of impaired perfusion. Hart and colleagues addressed this by reporting the evidence of preserved mitochondrial respiratory capacity in patients with early stage PAD, despite a reduction in perfusion (Hart et al., 2018). This implies that impaired mitochondrial capacity may be a sequela of limited convective O_2_ delivery. Nevertheless, considering that both of these factors (reduced perfusion and impaired utilization) would independently decrease the O_2_ pressure gradient between the capillary bed and muscle fiber, it should not be surprising that early onset O_2_ kinetics have also been reported to be slower in PAD patients during steady‐state exercise (Bauer et al., 2004; Bauer et al., 2007). However, despite slower onset kinetics, the magnitude of skeletal muscle desaturation is augmented during graded submaximal and fatiguing work in PAD (Luck et al., 1985). This indicates that O_2_ utilization increases relative to O_2_ supply, reflecting an elevated metabolic perturbation which likely contributes to the exaggerated exercise pressor reflex observed in these patients (Miller et al., 1985; Muller et al., 2015; Ross et al., 2017).

Considering the role of the exercise pressor reflex in augmenting peripheral blood flow during physical activity (Amann et al., 2011; Amann et al., 2015), it is reasonable to suspect that an exaggerated pressor reflex would increase convective O_2_ delivery to the affected limbs of PAD patients during presymptomatic exercise. Ultimately, this would imply that the exaggerated exercise pressor reflex observed in PAD is part of a compensatory mechanism that facilitates O_2_ transport at light intensities. This hypothesis would be supported by a strong positive relationship between the magnitude of the exercise pressor reflex and the change in O_2_ transport during exercise. Accordingly, this project aimed to compare O_2_ transport in the affected limbs of PAD patients to healthy controls (CON) during light intensity presymptomatic plantar flexion. Through the use of quantitative magnetic resonance imaging (MRI), we were able to simultaneously record perfusion and an index of oxygenation (T_2_*) from the medial gastrocnemius (MG), as well as venous oxygen saturation (S_v_O_2_). We expected that a rapid decrease in tissue oxygenation would evoke an exaggerated pressor response in PAD patients, which would normalize tissue perfusion and increase O_2_ extraction. We also expected that the exercise pressor response would be positively correlated with changes in O_2_ transport.

## Methods

### Subjects and study design

All data were collected at the Penn State Hershey Clinical Research Center (CRC) in the Clinical and Translational Science Institute and at the Center for Nuclear Magnetic Resonance Research (CNMRR), and all experiments were approved by the Penn State Hershey College of Medicine Internal Review Board (IRB#00005331). A total of 15 subjects were recruited for participation in this study (*n* = 8 PAD, *n* = 7 healthy). However, one PAD patient's data were excluded as an outlier (symptoms inconsistent with all other patients), resulting in a final sample size of 14 (Table 1). Participation included one to two visits to the lab. Specifically, patients with unilateral PAD, defined by an ankle brachial index (ABI) <0.9 in one leg (*n* = 2), completed a single visit in which their symptomatic leg was imaged with MRI during graded plantar flexion exercise. Patients with bilateral PAD, defined by an ABI <0.9 in both legs (*n* = 5), completed a second visit in order to image the other (also symptomatic) leg during the same exercise. Data from each patient were then averaged between both visits before final analysis, providing a single (mean) score for both symptomatic legs. Each PAD patient was also matched (within a 10% margin of error) for age and BMI to a healthy control subject, and each control subject performed the same exercises using the same leg(s) as their matched PAD patient. The patients involved in this study were taking a variety of prescribed medications, including Plavix (*n* = 2), Aspirin (*n* = 5), Statins (*n* = 7), Antihypertensives (*n* = 4), β‐blockers (*n* = 2), and drugs specifically labeled for PAD (i.e., Cilostazol and Pentoxifylline; *n* = 3). One healthy control subject was also prescribed a low‐dose Aspirin regimen. Subjects were not instructed to withhold medications prior to data collection. Many of these patients had also received prior interventions for restoring limb blood flow, including angioplasty (*n* = 3), stent placement (*n* = 3), and femoral artery bypass (*n* = 3). However, all patients had experienced reocclusion/stenosis, as indicated by an ABI <0.9.

**Table 1:** Subject demographics

	Healthy (*1 female*)	PAD (*1 female*)
*n*	7	7
Age	66 ± 7	66 ± 6
Height (cm)	174.8 ± 7.6	172.7 ± 10.2
Weight (kg)	82.4 ± 12.4	86.3 ± 10.1
BMI (kg/m^2^)	26.6 ± 2.3	28.1 ± 2.3
ABI‐right	1.10 ± 0.1	0.78 ± 0.2*
ABI‐left	1.05 ± 0.1	0.56 ± 0.2*
Resting SBP	133.4 ± 10.7	133.2 ± 10.4
Resting DBP	81.2 ± 9.1	79.2 ± 11.4
Resting HR	67.1 ± 6.2	79.1 ± 9.9#
End exercise pain	0.0 ± 0.0	5.5 ± 2.9*
End exercise RPE	12.4 ± 1.4	14.5 ± 2.2#
Achieved workload (kg)	10.0 ± 0.0	8.5 ± 1.9

^#^ *P* < 0.05,

* *P* < 0.01.

### Experimental protocols

**Figure 1:**
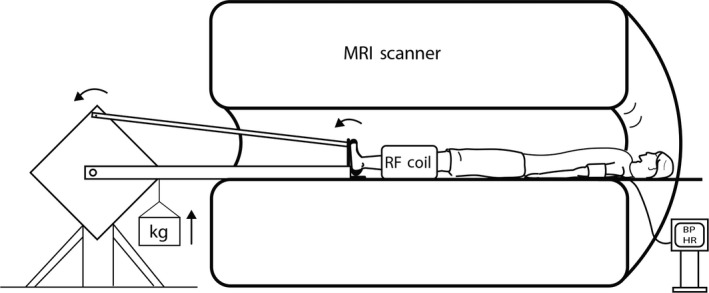
A depiction of the non‐ferromagnetic dynamic plantar flexion device that permitted exercise to be performed inside the bore of a 3T MRI scanner. Blood pressure was also monitored during exercise using a MRI‐compatible automated brachial blood pressure monitor

Each subject performed a graded plantar flexion protocol inside the bore of a 3T MRI scanner (Siemens Healthineers, Erlangen, Germany). This was accomplished using a custom built non‐ferromagnetic device, as depicted in Fig. 1. Once instrumented, subjects rested for 2 minutes while baseline data were collected. The actual time was 120.25 seconds (2.004 minutes), which allowed for 37 complete MRI scans. This was immediately followed by a graded plantar flexion protocol that began at a 2 kg workload (30 contractions/minute) and increased by 2 kg every 2 minutes until fatigue, or until the completion of 10 kg. Subjects rested during the final 10 seconds of each 2‐minute stage while their leg was imaged. This exercise protocol was designed to maximize the period of presymptomatic exercise in PAD patients while also eliciting a typical cardiovascular response to exercise in healthy controls.

### Measurements

Upon arrival, all subjects' anthropometric and resting data were collected at the Penn State Hershey CRC. These data included resting blood pressure, heart rate (HR), height, weight, and ABI in both legs (Table 1). After this, subjects were escorted to the CNMRR facility by a member of the research team.

All experiments were performed on a Siemens 3T PrismaFit scanner with a 15 channel knee coil (Quality Electrodynamics, Mayfield, Ohio) used for both signal transmission and reception. MRI data were acquired with a custom implementation of the PIVOT technique (Englund et al., 2013). The sequence repetition time was 3.25 seconds, leaving 250 ms within the 10‐second rest period for the MRI operator to initiate the PIVOT sequence. Within each 3.25 seconds sequence, both arterial spin labeling (ASL) and multi‐echo gradient‐echo (mGRE) images were acquired for calculation of tissue perfusion maps, T_2_* maps, and S_0_ maps of the MG, as well as venous oxygen saturation. The MG was specifically selected for analysis due to its primary involvement in straight‐knee (180°) plantar flexion, which has been confirmed with electromyography (EMG) (Signorile et al., 2002). For clarity, the ASL image quantifies tissue perfusion by “tagging” the inflow of blood in the microvascular compartment of a selected region of interest (ROI) (Raynaud et al., 2001; Englund et al., 2013), and was acquired with a single‐shot echo‐planar imaging (EPI) readout, echo time (TE) 9.6 ms, matrix 64 × 64, resolution 2.75 × 2.75 × 8.0 mm, partial Fourier factor 5/8, excitation flip angle 90°, and a spectral‐spatial pulse (Meyer et al., 1990) to suppress the signal from fat. The unit of measurement for perfusion measured by the ASL technique is milliliter of blood per minute per 100 g of muscle. However, we do not actually measure tissue composition. Rather, this is estimated from the area of the ROI. While the ASL sequence provides a measure of muscle perfusion, T_2_* is a measure of relative oxygen saturation within the muscle vascular compartment. This signal is relatable to the Blood‐Oxygen‐Level‐Dependent (BOLD) signal (Muller et al., 2016), and is inherently sensitive to changes in deoxyhemoglobin (as deoxyhemoglobin increases, the signal intensity decreases). The BOLD effect is influenced by a combination of blood flow and volume (or perfusion), O_2_ turnover, and venous mobilization. We supplemented this by also calculating S_0_, a measure of total water content, and presenting T_2_* as normalized to S_0_ (T_2_*/S_0_; labeled as T_2_*norm). In contrast to T_2_*, S_v_O_2_ quantifies venous oxygen saturation through a phase‐contrast technique. Each of these measurements (T_2_*, S_v_O_2_, and S_0_) were collected from the same mGRE sequence, which was acquired with a five echo monopolar readout, TE = [4.46,7.73,13,19,25] ms, steady‐state repetition time (TR) of 27.5 ms, matrix 92 × 92, resolution 1.1 × 1.1 × 10 mm, and excitation flip angle 15 degrees. The GRAPPA (generalized autocalibrating partially parallel acquisitions) technique (Griswold et al., 2002) was applied to the mGRE image acquisition to reduce the number of acquired k‐space lines from 92 to 56. Data were reconstructed and analyzed with custom Matlab programs (Mathworks, Natick, MA), following the techniques outlined by Englund and colleagues (Englund et al., 2013). Preliminary experiments confirmed that this customized PIVOT sequence was sensitive to group differences during reactive hyperemia. To calculate our MRI variables, all data were first averaged over the final 10 scans of baseline. Next, T_2_*, S_0_, and S_v_O_2_ were averaged over the final two scans collected at the 2 kg and 4 kg workloads, which were then used to calculate the absolute change from baseline (a.k.a. a delta score). The first mGRE scan was discarded, as one full scan was required for the tissue to reach the appropriate magnetized state (indicated by a steady‐state signal at baseline; data not shown). Similarly, the ASL technique required two full scans to reach a steady‐state value at baseline, and therefore perfusion was calculated as a change score from baseline to the third scan at each workload.

In addition to MRI data, systolic blood pressure (SBP), diastolic blood pressure (DBP), mean arterial pressure (MAP), and HR were recorded at every stage during exercise using an automated MRI‐compatible brachial blood pressure monitor (Invivo Corp., Gainesville, FL). These values were averaged between both visits in bilaterally diseased PAD patients, as well as their healthy controls prior to final analysis. Borg's Rating of Perceived Exertion (RPE) was also collected after the final achieved workload in each subject, as was leg pain via the Numeric Rating Scale (Jacox et al., 1994). As noted above, these data were averaged between visits for each bilateral PAD patient and their matched healthy controls, providing a single mean value for each subject.

### Statistical analysis

Based on an estimated effect size of 0.42, a power analysis indicated that a total of 12 subjects (six per group) would be required to reach significance with a desired power of 0.80 and significance accepted at *P* = 0.05. All power calculations were performed using G*Power 3.1 software (Faul et al., 2007). Once data were collected, subject characteristics were compared between groups using paired samples *t*‐tests, as were pain, RPE, and achieved workload at end‐exercise. Our primary outcome variables were then tested for normality using a Shapiro–Wilk test. Once the assumption of normality was satisfied, we tested for group by time interactions and main effects of group or time using a 2 (PAD vs. Controls) by 3 (Baseline, 2 kg, and 4 kg) repeated measures analyses of variance (RMANOVA). These included all MRI data, blood pressure, and HR. Any significant interactions or main effects were analyzed further with post‐hoc analyses using a Sidak correction. To provide a value that would reflect total O_2_ extraction, the “O_2_ Extraction Index” was calculated by the following equation:

$$O_{2}\;\text{Extraction Index}=\left(P_{t}-\;P_{0}\right)-\left(S_{t}-S_{0}\right).$$

where *P_t_* is the perfusion at the selected workload (i.e., 2 kg or 4 kg), *P_0_* is the perfusion at baseline, *S_t_* is the S_v_O_2_ at the selected workload, and *S_0_* is the S_v_O_2_ at baseline. This index treats tissue perfusion as an indirect measure of convective O_2_ delivery, and therefore assumes the difference between perfusion and venous O_2_ saturation to be an approximate index of O_2_ delivered to the muscle fiber. Similar to other MRI data, O_2_ extraction index was compared between groups using a 2 (PAD vs. controls) by 3 (Baseline, 2 kg, and 4 kg) RMANOVA, and any significant interactions or main effects were analyzed further with a Sidak post‐hoc correction. Lastly, the relationships between the change in perfusion and the pressor response (ΔMAP), as well as O_2_ extraction index and the pressor response were tested using a bivariate Pearson's correlation analysis. Significance was accepted at *P* = 0.05, and data are presented as mean ± standard error (SE). All statistical analyses were performed using SPSS version 25 (IBM Corp., Armonk, NY).

## Results

**Figure 2:**
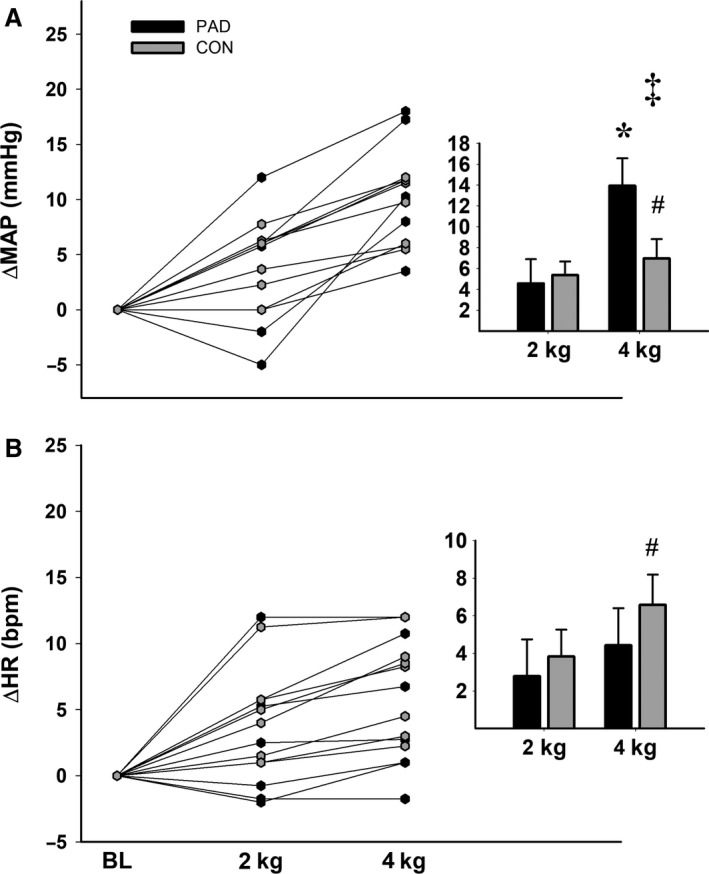
Changes in mean arterial pressure (A) and heart rate (B) compared between groups and across workloads. Raw data are presented on the left, and summary data are presented in the paneled figures. * indicates a significant difference from baseline in the PAD group, # indicates a significant difference from baseline in the CON group, and ‡ indicates a significant difference between groups. Alpha was set a priori at *P* ≤ 0.05*,* and all data are presented as mean ± SE

Of the seven PAD patients whose data were included in the final analysis, four were able to complete the entire exercise protocol. Of the subjects who did not complete the exercise protocol, one reached fatigue at 8 kg, and two reached fatigue at 6 kg. Therefore, we only analyzed data through the 4 kg workload, as this best characterized the presymptomatic period in our PAD sample. As expected, exercise required more perceived effort and elicited a higher pain response in PAD patients compared to healthy controls (Table 1). Exercise also elicited an augmented pressor response in PAD patients, as indicated in Fig. 2. Specifically, SBP (*F*_2,24_ = 5.10, *P* = 0.014) and MAP (*F*_2,24_ = 4.36, *P* = 0.024) both increased more at the 4 kg workload in PAD patients compared to controls (ΔMAP = +14 mmHg ± 4 and + 7 mmHg ± 2 [Fig. 2A] and ∆SBP = +23 mmHg ± 5 and +9 mmHg ± 2 in PAD and CON, respectively, all *P* ≤ 0.034). HR also increased in the control group at 4 kg (*F*_2,24_ = 10.43, *P* ≤ 0.001; ΔHR = +7 bpm ± 1, *P = *0.024; Fig. 2B), although no significant differences were observed between groups.

### MRI data

**Figure 3:**
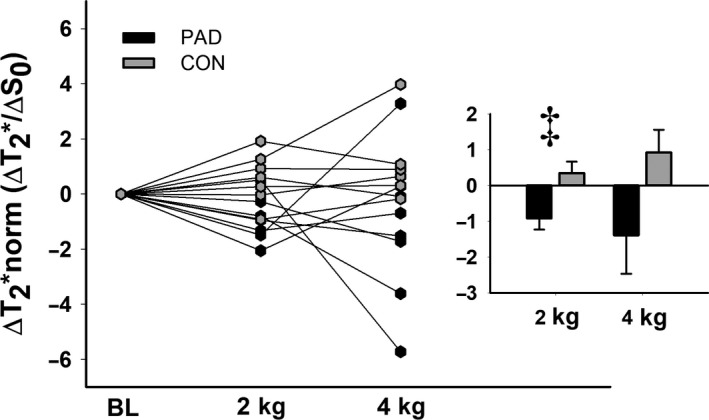
Changes in T_2_*norm compared between groups and across workloads. Raw data are presented on the left, and summary data are presented in the paneled figures. ‡ indicates a significant difference between groups. Alpha was set a priori at *P* ≤ 0.05*,* and all data are presented as mean ± SE

During exercise, MG T_2_* displayed a net, but nonsignificant decrease from baseline at the 2 kg workload in the PAD group compared to healthy controls (*F*_1,12_ = 2.27, *P* = 0.15). This was consistent when T_2_* was represented as a percent change. However, it is worth noting that the relaxation time of T_2_* is primarily driven by the relative quantities of oxygenated and deoxygenated hemoglobin in the intravascular space (Sanchez et al., 2010). Therefore, to acquire a more independent measure of muscle tissue oxygenation changes relative to metabolic activity, we normalized changes in T_2_* to changes in S_0_ (an index of free water content). This calculated variable was labeled T_2_*norm (ΔT_2_*/ΔS_0_) and was tested using the same statistical analyses listed for other MRI data. Results indicated that T_2_*norm was significantly lower in the PAD group at 2 kg compared to healthy controls (*F*_1,12_ = 17.04, ΔT_2_*norm = −0.91 Δms/ΔAU ± 0.3 and 0.57 Δms/ΔAU ± 0.3 in PAD and CON, respectively, all *P* ≤ 0.008; Fig. 3). However, no significant differences were observed between groups at 4 kg (ΔT_2_*norm = −1.3 Δms/ΔAU ± 1.0 and 0.94 Δms/ΔAU ± 0.5 in PAD and CON at 4 kg, respectively, *P* = 0.077).

**Figure 4:**
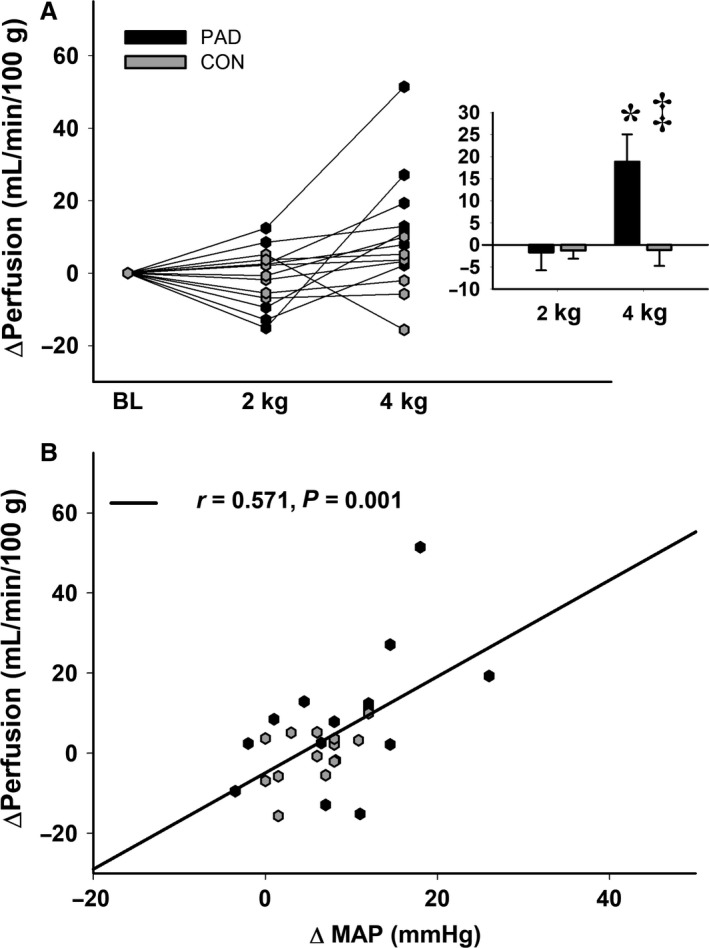
Changes in perfusion (A) compared between groups and across workloads. Raw data are presented on the left, and summary data are presented in the paneled figures. * indicates a significant difference from baseline in the PAD group, and ‡ indicates a significant difference between groups. Tile (B) illustrates the relationship between perfusion and the blood pressure response to exercise. Alpha was set a priori at *P* ≤ 0.05, and all data are presented as mean ± SE

In contrast, perfusion of the MG was not different between groups at 2 kg, but increased far more in the PAD group at 4 kg compared to healthy controls (*F*_2,24_ = 6.71, ΔPerf = +18.8 mL/min/100g ± 6.2 and −0.21 mL/min/100g ± 3.2 at 4 kg in PAD and CON, respectively, *P* ≤ 0.026; Fig. 4A). Although PAD is a disease characterized by a perfusion limitation, we believe that the exaggerated exercise pressor reflex observed in PAD altered the perfusion pressure to the affected limbs during very light, presymptomatic work. This notion is supported by a statistically significant and positive relationship between the change in perfusion and the change in MAP (*r* = 0.571, *P* = 0.001; Fig. 4B); although a direct cause and effect relationship remains to be established.

**Figure 5:**
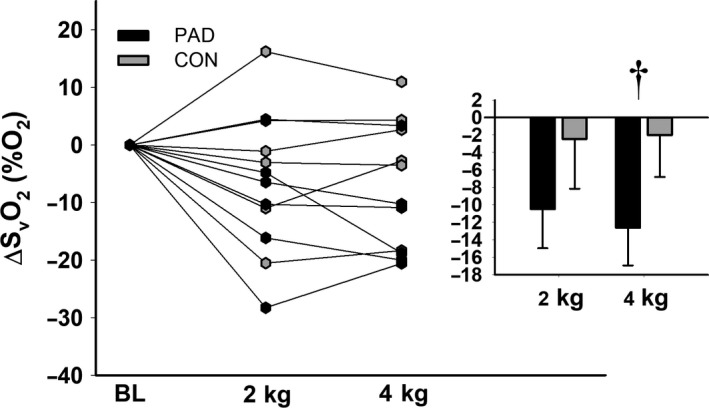
Changes in S_v_O_2_ compared between groups and across workloads. Raw data are presented on the left, and summary data are presented in the paneled figures. † indicates a significant difference from baseline in the entire sample. Alpha was set a priori at *P* ≤ 0.05, and all data are presented as mean ± SE

**Figure 6:**
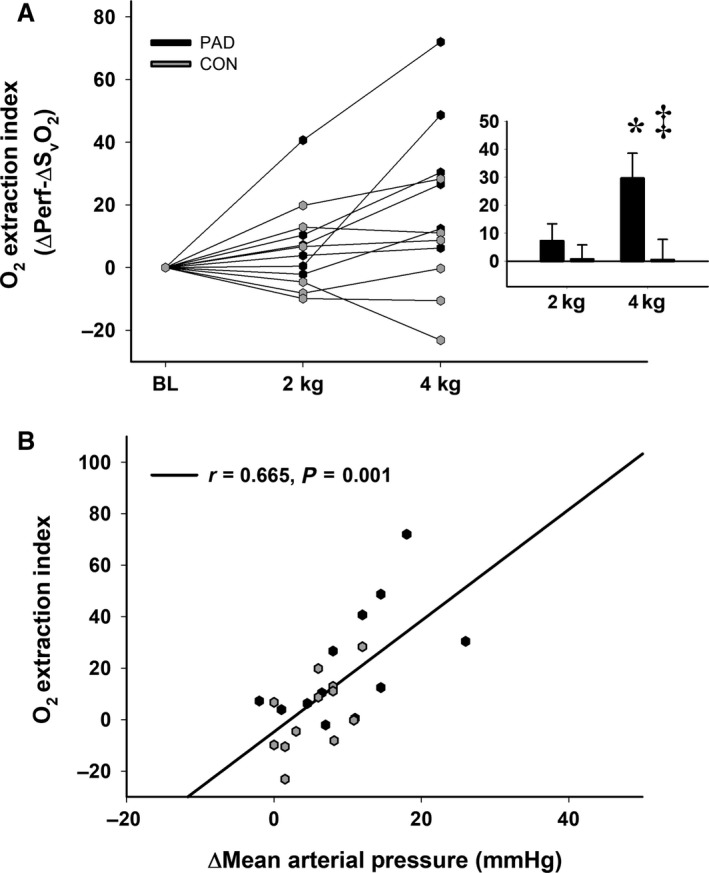
Changes in O_2_ extraction index (A) compared between groups and across workloads. Raw data are presented on the left, and summary data are presented in the paneled figure. Tile (B) illustrates the relationship between O_2_ extraction index and the blood pressure response to exercise. * indicates a significant difference from the 2 kg workload in the PAD group and ‡ indicates a significant difference between groups. Alpha was set a priori at *P* ≤ 0.05, and all data are presented as mean ± SE

Results also indicated a significant effect of time for S_v_O_2_, such that S_v_O_2_ decreased at 4 kg in both groups (*F*_2,24_ = 4.55, ΔS_v_O_2_ = −13% ± 4 and −2% ± 4 at 4 kg in PAD and CON, respectively, *P* ≤ 0.04; Fig. 5). A significant interaction was also observed for the O_2_ extraction index (Δperf‐ΔS_v_O_2_), explained by a significantly greater increase in the O_2_ extraction index in the PAD group at 4 kg compared to healthy controls (*F*_2,24_ = 5.49, ΔO_2_ extraction index = 32.72 AU ± 9.9 and 2.35 AU ± 7.3 in PAD and CON, respectively, all *P* ≤ 0.049; Fig. 6A). Lastly, a Pearson's correlation analyses indicated that the O_2_ extraction index was significantly and positively correlated to the pressor response (Fig. 6B). This relationship persists when each group is tested independently (PAD: *r* = 0.603, *P* = 0.038; CON: *r* = 0.600, *P* = 0.039).

## Discussion

This study aimed to identify the potential role of the exercise pressor reflex in mediating O_2_ transport during presymptomatic work in PAD patients. Our data collectively suggest that O_2_ transport is augmented during graded presymptomatic exercise in these patients, and that this may be partially due to an exaggerated exercise pressor reflex. Specifically, it appears that the relative increase in perfusion to the affected limbs of PAD patients is greater at the second (4 kg) workload compared to controls (Fig. 4A), and this is significantly correlated with changes in MAP. Therefore, it is reasonable to suspect that the exaggerated pressor response to exercise in the PAD group, which was also observed at the second (4 kg) workload, consequently increased the perfusion pressure to the affected limb. Furthermore, S_v_O_2_ significantly decreased in both groups with increasing workload, suggesting that O_2_ extraction was much greater at 4 kg in PAD patients. This is based on the notion that perfusion functions as an indirect estimate of convective O_2_ delivery. It is also important to note that these data do not imply that PAD patients utilize more O_2_, but rather, the relative increase in O_2_ extraction was greater in PAD patients compared to healthy controls. Nevertheless, the increase in O_2_ extraction was well correlated with the magnitude of the pressor response, supporting the hypothesis that the exaggerated exercise pressor reflex may be part of a compensatory mechanism that facilitates presymptomatic O_2_ transport in PAD patients. With that in mind, we present some plausible explanations for this observation.

### Plausible explanations

The exaggerated exercise pressor reflex in PAD is primarily attributed to an increased stimulation of mechanosensitive Group III and metabosensitive Group IV muscle afferents (Drew et al., 2013; Stone et al., 2015). This is due, in part, to a greater responsiveness of these fibers to controlled levels of external input, such as capsaicin (Tsuchimochi et al., 2010) and muscle stretch (Kempf et al., 2018). Additionally, oxidative stress and stimulation of acid‐sensing ion channels (by changes in pH balance) have been reported to play significant roles in evoking the exaggerated pressor response in both human and animal models of PAD (Tsuchimochi et al., 2011; Muller et al., 2012; Farrag et al., 2017; Harms et al., 2017; Xing et al., 2018). Therefore, the initial decrease in muscle oxygenation observed in our study, and others (Ledermann et al., 2006), would likely contribute to the exaggerated stimulation of these Group III and IV afferents via the aforementioned mechanisms. Ultimately, this would initiate the cascade by which perfusion pressure would be increased to the affected limb(s) at these very light workloads. Similarly, this same decrease in tissue oxygenation would decrease the partial pressure of O_2_ within the skeletal muscle, effectively increasing the passive O_2_ diffusion gradient between the capillary and skeletal muscle fiber, which would be potentiated by the concurrent increase in tissue perfusion and convective O_2_ delivery.

To add to this, muscular efficiency may also be blunted in PAD, resulting in an increased O_2_ requirement for a controlled amount of absolute work. Indeed, prior investigations have reported exercise training‐related improvements in walking economy (a functional measure of muscular efficiency) in PAD patients, which were associated with improved functional outcomes (Gardner et al., 2002; Ritti‐Dias and Wolosker, 2010; Crowther et al., 2012). Similarly, prior reports also indicate an increased rate of skeletal muscle degeneration (Makitie and Teravainen, 1977) and fibrosis (Ha et al., 2016) in PAD. Therefore, a reduction in muscle quality, or even myofiber quantity could lead to an increased relative oxygen cost at the same absolute submaximal workload. This would result in a greater reliance on non‐oxidative metabolism, evoking a more robust pressor response. In contrast, if exercise was prescribed based on relative oxygen cost (%Vo_2_max), the pressor response and subsequent increase in perfusion may be much more comparable between groups. Furthermore, the mitochondriopathy associated with PAD, as outlined by Pipinos and colleagues (Pipinos et al., 2007), would likely result in an increased vulnerability of slow‐oxidative Type I muscle fibers to myopathy. However, work by Charles and colleague (Charles et al., 2017) suggests that oxidative muscle fibers are better protected from ischemia‐reperfusion injury due to their elevated antioxidative properties. Clearly, more work is needed to better define the alterations in muscle phenotype during the progression of PAD.

It is important to note that, at first glance, our results may seem to contradict previous reports from Bauer and Colleagues (Bauer et al., 2004; Bauer et al., 2004; Bauer et al., 2007). Specifically, we report an augmentation of O_2_ extraction in PAD patients while Bauer and colleagues report a slower O_2_ uptake response. However, we do not dispute the conclusions of these studies, nor do we argue against the concept of a blunted O_2_ consumption curve in PAD patients. Instead, we provide evidence that O_2_ transport increases more in PAD patients as a result of graded presymptomatic exercise, and we do not address the early onset phase at each workload. In fact, a slower O_2_ consumption response during steady‐state exercise would contribute to a larger O_2_ deficit, which would increase the reliance on non‐oxidative metabolism across each workload. Considering that this would increase the metabolite accumulation and contribute to the exaggerated pressor response in PAD patients, these data actually support our findings.

### Implications

Understanding the mediators of O_2_ transport during these very light intensity workloads in PAD patients can be important for a number of reasons. First, these very early changes in O_2_ transport may be a compensatory mechanism that precedes the subsequent truncation of O_2_ consumption at higher intensities. If true, then this could have important mechanistic implications for the development of O_2_ consumption limitations during the progression of PAD. Second, these results may suggest that the exaggerated exercise pressor reflex observed in PAD patients could actually facilitate some activities of daily living. For instance, the workloads used in this study may be equivalent to pushing the gas or brake pedals in a car, walking down a set of steps, or even transitioning between standing and sitting. Without an elevated pressor response, the perfusion pressure to the affected muscle may not be sufficient to maintain an appropriate level of O_2_ delivery. This would result in accelerated muscular fatigue, and ultimately a loss of function.

### Limitations

As is the case with any study, this project is subject to certain limitations. First, the ASL method of acquiring perfusion is subject to a “low‐flow” limitation. That is, perfusion as measured by ASL is not well defined without increases in flow rate (Raynaud et al., 2001). Therefore, we cannot say that perfusion was different between groups at baseline, nor can we assume that perfusion did not increase at all in the control group. Instead, perfusion may have increased, but just not to the level that is detectible by ASL. Because of this, we were limited to reporting change scores for our variables. However, despite this limitation, the relative perfusion responses were very consistent within our PAD and control groups. Also, there is an inherent heterogeneity in most PAD samples, and ours is no different. For example, we present a combination of unilaterally (*n* = 2) and bilaterally (*n* = 5) diseased PAD patients, which may introduce some variability in the responses to exercise. Because of this, we present all individual data in each of our figures. In addition, interindividual differences in muscle architecture of the triceps surae may also have introduced some heterogeneity in recruitment patterns between the MG, lateral gastrocnemius, and soleus muscles (Lauber et al., 2014). Despite this, we are still confident that the MG was the primary muscle recruited during the straight‐leg plantar flexion protocol used in this study (Signorile et al., 2002). Another limitation is that the exercise protocol used in this study was not relative to functional capacity. Instead, we used a graded exercise protocol comprised of absolute workloads. While this may introduce more variability to our exercise responses, we believe absolute workloads are more easily relatable to activities of daily living. Nevertheless, we intend to expand our exercise protocol in future studies.

### Future directions

As noted above, the exercise protocol employed in this study consisted of graded absolute workloads. To better define the mechanisms of O_2_ transport during very light presymptomatic work in PAD patients, we intend to compare responses between PAD patients and heathy controls using a combination of absolute and relative intensities. This will require a functional capacity assessment using plantar flexion exercise, which is something we are currently developing. We are also developing an updated quantitative MRI sequence that will provide measures of arterial O_2_ saturation as well as arterial blood flow, as outlined by Englund and colleagues (Englund et al., 2018). By employing the Fick principle, we will be able to improve our interpretation of O_2_ consumption. Ultimately, we hope to better define the O_2_ response curve in PAD patients across a wide range of absolute and relative exercise intensities.

### Conclusions

Our data suggest that O_2_ transport is augmented in the MG of PAD patients during graded presymptomatic exercise. We postulate that this may be mediated, in part, by an exaggerated exercise pressor reflex. While these results may have implications for how O_2_ transport mechanisms are altered during the progression of PAD, more research is needed to better define the presymptomatic O_2_ response curve in these patients.

## Funding information

This project was supported by NIH P01 HL134609 (Sinoway) and UL1 TR002014 (Sinoway).

## Conflict of interest

The authors have no conflict of interest to disclose.
